# Labile Heme and Heme Oxygenase-1 Maintain Tumor-Permissive Niche for Endometriosis-Associated Ovarian Cancer

**DOI:** 10.3390/cancers14092242

**Published:** 2022-04-29

**Authors:** Jonathan L. Hecht, Monika Janikova, Reeham Choudhury, Fong Liu, Giacomo Canesin, Lubica Janovicova, Eva Csizmadia, Elisa M. Jorgensen, Katharine M. Esselen, Peter Celec, Kenneth D. Swanson, Barbara Wegiel

**Affiliations:** 1 Department of Pathology, Beth Israel Deaconess Medical Center, Harvard Medical School, 330 Brookline Ave, Boston, MA 02215, USA; jlhecht@bidmc.harvard.edu; 2 Department of Surgery, Division of Surgical Sciences, Beth Israel Deaconess Medical Center, Harvard Medical School, Boston, MA 02215, USA; mjanikov@bidmc.harvard.edu (M.J.); reehamchoudhury444@gmail.com (R.C.); giacomo.canesin@gmail.com (G.C.); lubica.janovicova@gmail.com (L.J.); ecsizmad@bidmc.harvard.edu (E.C.); 3 Faculty of Medicine, Institute of Molecular Biomedicine, Comenius University in Bratislava, 814 99 Bratislava, Slovakia; petercelec@gmail.com; 4 Department of OB/GYN, Beth Israel Deaconess Medical Center, Harvard Medical School, Boston, MA 02115, USA; fongwliu@gmail.com (F.L.); ejorgen1@bidmc.harvard.edu (E.M.J.); kesselen@bidmc.harvard.edu (K.M.E.); 5 Greater Baltimore Medical Center, 6569 Charles Street, Towson, MD 21204, USA; 6 Vor Biopharma, 100 Cambridgepark Dr, Suite 400, Cambridge, MA 02140, USA; 7 Department of Neurology, Beth Israel Deaconess Medical Center, Harvard Medical School, Boston, MA 02215, USA; kswanson@bidmc.harvard.edu

**Keywords:** endometriosis, ovarian cancer, labile heme, hemopexin, heme oxygenase-1

## Abstract

**Simple Summary:**

Endometriosis is an immediate precursor lesion of ovarian clear cell cancer (OCCC) and is heavily infiltrated by functionally impaired immune cells including macrophages. A key role of macrophages in endometrial lesions is to process significant amount of heme released from shedding of the endometrium during menstruation by the activity of heme oxygenase-1 (HO-1, *Hmox1*). Heme is a pro-inflammatory molecule that induces oxidative stress. We propose that heightened oxidative stress, inflammation, and the presence of iron-containing molecule such as heme in the immune niche due to altered heme metabolism may be a driver of OCCC. This study contributes to better understanding of the mechanisms of malignant progression of OCCC.

**Abstract:**

Endometriosis, a painful gynecological condition accompanied by inflammation in women of reproductive age, is associated with an increased risk of ovarian cancer. We evaluated the role of peritoneal heme accumulated during menstrual cycling, as well as peritoneal and lesional macrophage phenotype, in promoting an oncogenic microenvironment. We quantified the heme-degrading enzyme, heme oxygenase-1 (HO-1, encoded by *Hmox*1) in normal peritoneum, endometriotic lesions and endometriosis-associated ovarian cancer (EAOC) of clear cell type (OCCC). HO-1 was expressed primarily in macrophages and increased in endometrioma and OCCC tissues relative to endometriosis and controls. Further, we compared cytokine expression profiles in peritoneal macrophages (PM) and peripheral blood mononuclear cells (PBMC) in women with endometriosis *versus* controls as a measure of a tumor-promoting environment in the peritoneum. We found elevated levels of HO-1 along with IL-10 and the pro-inflammatory cytokines (IL-1β, IL-16, IFNγ) in PM but not in PBMC from endometriosis patients. Using *LysM-Cre:Hmox1^flfl^* conditional knockout mice, we show that a deficiency of HO-1 in macrophages led to the suppression of growth of ID8 ovarian tumors implanted into the peritoneum. The restriction of ID8 ovarian tumor growth was associated with an increased number of Mac3^+^ macrophage and B cells in *LysM-Cre:Hmox1^flfl^* mice compared to controls. Functional experiments in ovarian cancer cell lines show that HO-1 is induced by heme. Low levels of exogenous heme promoted ovarian cancer colony growth in soft agar. Higher doses of heme led to slower cancer cell colony growth in soft agar and the induction of HO-1. These data suggest that perturbation of heme metabolism within the endometriotic niche and in cancer cells themselves may be an important factor that influences tumor initiation and growth.

## 1. Introduction

Endometriosis is an inflammatory disease characterized by lesions of endometrial-like tissue outside the uterus (pelvic peritoneum and ovaries) that is associated with pain and infertility [1]. Although the epithelium in these lesions often carries cancer driver mutations and can exhibit clonal growth, the lesions are considered benign, as they remain confined to their site of implantation without rapid proliferation or metastasis [2,3]. However, implants of endometriosis, especially when involving the ovary as an endometriotic cyst, can, rarely, transform into endometriosis-associated ovarian cancer (EAOC). These cancers are often of the less common clear cell subtype (OCCC, ovarian clear cell carcinoma), [4] for which the triggers of transformation and growth are poorly understood. 

The lack of a consistently detected combination of cancer driver mutations or histologic precancerous changes in endometriotic lesions suggests that factors in the microenvironment or epigenetic changes may play a role in oncogenesis. We hypothesize that EAOC is driven by heightened oxidative stress, inflammation and presence of iron-containing molecules, such as heme, [5,6] that are prominent in endometriosis due to cyclic bleeding with the menstrual cycle. Heme and ferritin levels are elevated in the peritoneum of patients with endometriosis, which may also reflect disturbed iron metabolism in the peritoneal cavity [7]. 

Free heme is a pro-inflammatory molecule that induces oxidative stress via the Fenton reaction, generating reactive oxygen species (ROS). Free heme is scavenged by hemopexin (Hx) and its receptor on myeloid cells, CD91/LRP, followed by heme degradation by the activity of heme oxygenases (HO, HO-1 and HO-2) [8] that generate carbon monoxide, biliverdin, and iron. Heme metabolism is a critical mediator in maintaining cellular homeostasis and is orchestrated by tissue-associated immune cells, particularly macrophages [6,9]. Disruption in heme homeostasis is a common feature of cancer [10]. High expression of nuclear HO-1 in cancer cells and tumor-associated macrophages correlates with poor prognosis of prostate, lung, and skin cancers, and leukemias [11,12,13,14] and has been described in vitro in responses to oxidative stressors and hypoxia [15]. 

The exact role of HO-1 and mechanisms of action in endometriosis and EAOC are poorly understood [16,17]. Excessively high levels of heme and iron during menstrual cycling, and attenuated levels of Hx, may lead to oxidative modifications of lipids and proteins as well as DNA damage, ultimately resulting in fibrosis in the peritoneum of women with endometriosis [5]. We propose that heme and heme metabolism trigger an alteration in peritoneal and lesional macrophage phenotype that is permissive to cancer. 

The heme scavenger, Hx, is reduced in the peritoneal fluid of patients with endometriosis [18]. Hx has not been studied in EAOC, but is thought to play a role in cancer survival. Hx expression is highly correlated with poor five-year overall survival rate in hepatocarcinoma (HCC) [19]. Fucosylated Hx is a valuable biomarker for HCC [20]. Hx was also identified as a potential serological biomarker for lung adenocarcinoma [21]. We have recently shown that deletion of Hx in the tumor niche or treatment with heme promotes prostate cancer metastasis via mechanisms involving a direct interaction of labile heme with secondary structures of DNA, G-quadruplexes, in genes such as c-MYC [10]. 

In this study, we determined the tissue expression of HO-1 and Hx in peritoneal biopsies from women with endometriosis, controls and in tissue from endometriosis-associated ovarian clear cell cancer. We hypothesized that high levels of heme and, thus, induction of HO-1 in endometriosis may slow the growth and evolution of these cancers. This is consistent with the common presentation at low stage, and long time to recurrence of OCCC. 

We also compare RNA expression of these genes between endometriosis and paired samples of normal eutopic endometrium using in-silico analysis of published data. Expression is generally confined to stromal macrophages and, in cases of carcinoma, the neoplastic epithelium. Using functional studies in ovarian cancer cell lines, we found that low levels of heme are insufficient to induce HO-1, but promote cancer growth, while higher heme levels suppress growth and induce HO-1. Using conditional HO-1 knockout mice, we found that HO-1 in macrophages is also critical for the growth of grafted peritoneal ovarian cancer cells. Using peritoneal macrophages isolated from women undergoing surgery, we demonstrate that women with endometriosis have an M2-like cytokine profile, when compared to control patients and matched peripheral blood monocytes. Our data suggest that endometriosis-associated heme-induced HO-1 may be a key regulator of EAOC progression.

## 2. Material and Methods

### 2.1. Patient Material

All studies were approved by the IRB committee at the BIDMC. 

Fresh specimens: Fresh PM, PBMC and peritoneal biopsies were collected from patients with endometriosis (*n* = 9) and controls (*n =* 5) following the IRB protocol. Control group included women undergoing surgery due to: genetic susceptibility to cancer (*n* = 3), uterine fibroids (*n* = 1) or lesion on cervix (*n* = 1). 

Peritoneal biopsies of endometriosis and PM were harvested by washing the peritoneal cavity with 50–100 mL saline. The PM pellets were obtained by centrifuging washes at 1600× rpm for 5 min at room temperature (RT). Supernatants were transferred into 50 mL falcon tubes and stored at −80 °C. Pellets were resuspended in 1 mL 1× PBS (Thermo Fisher Scientific, Waltham, MA, USA) and centrifuged once at 1600× rpm for 5 min at RT. 

Blood was collected in the BD Vacutainer^®®^ Plus Venous Blood Collection Heparin Tube (BD Biosciences, Franklin Lakes, NJ, USA). PBMC were harvested using Ficoll-PaqueTM PLUS (GE Healthcare, IL, USA) density gradient centrifugation protocol. Blood was diluted 1:1 with 1× PBS. Furthermore, 15 mL of Ficoll-PaqueTM PLUS at RT was placed in 50 mL falcon tube and layered in 1:1 ratio with blood/1xPBS mixture using a transfer pipette to avoid mixing blood and Ficoll-PaqueTM PLUS phases. Samples were centrifuged at 400× g and 20 °C for 30 min selecting acceleration/deceleration rates 9/0. Using the transfer pipette, the mononuclear layer (PBMC) was carefully removed to a new tube and 3 volumes of 1x PBS were added to the samples. Samples were centrifuged at 500× g and 20 °C for 10 min. Pellets were stored at −80° until further analysis. 

Archival samples: All cases of endometriosis-associated clear cell ovarian carcinoma with the presence of cancer-adjacent endometriosis were pulled from 1998–2006 pathology archives, which included 17 cases. Endometrioma, endometriosis cases and hernia sac cases (*n* = 5 each) were from the 2019 pathology archives at BIDMC. The medical record was reviewed to determine the following patient characteristics: age and stage at diagnosis, adjuvant treatment (chemotherapy and/or radiation), disease status at the time of last-known contact, disease-free interval at the time of last-known contact. The formalin-fixed, paraffin-embedded issue blocks were sectioned and stained for HO-1, Hx, and CD45. The presence and intensity of protein staining was evaluated by both stain intensity and proportion of positive-staining cells. The intensity scale is as follows: 0 (no pigmentation), 1 (light yellow), 2 (buff), and 3 (brown). The percentage of positive cells was assessed by high power field: 0 (<5% chromatic cells), 1 (5–25% chromatic cells), 2 (26–50% chromatic cells), 3 (51–75% chromatic cells), and 4 (>75% chromatic cells). Scores for each pathology specimen was calculated and compared between the tissues using Pearson correlation. Here, p < 0.05 will be considered as statistically significant. Clinical characterization of patients is provided in Supplementary Table S1.

### 2.2. IHC and Analysis

Tissue samples were formalin fixed followed by paraffin embedding, and immunostaining of 5 μm sections was performed as previously described [22]. The following antibodies were used for human material: Hx (Abcam, Cambridge, MA, USA), HO-1 (Enzo Life Sciences, Farmingdale, NY, USA), and CD45 (BD Biosciences, Franklin Lakes, NJ, USA) and for mouse tissue staining: Mac-3 (BD Biosciences, Franklin Lakes, NJ, USA) and CD45R/B220 (BD Biosciences, Franklin Lakes, NJ, USA). 

Tissues were de-paraffinized and processed for antigen retrieval using high pressure cooking in citrate buffer. Sections were then blocked for 30 min in 7% horse serum (Vector Laboratories, Burlingame, CA, USA). Primary antibody was then applied to the sections overnight at 4 °C. The following day, sections were incubated with biotin-labeled secondary antibody (Vector Laboratories, Burlingame, CA, USA) for 1 h at room temperature, followed by VECTASTAIN Elite ABC kit and detection with ImmPACT DAB (Vector Laboratories, Burlingame, CA, USA). All images were captured using a Nikon Eclipse E600 microscope (Nikon Instruments, Melville, NY, USA). 

The presence and intensity of protein staining was evaluated by both staining intensity and percentage of positive cells. The intensity scale was as follows: 0 (no pigmentation), 1 (light yellow), 2 (buff), and 3 (brown). The percentage of positive cells was assessed by high power field: 0 (<5% chromatic cells), 1 (5–25% chromatic cells), 2 (26–50% chromatic cells), 3 (51–75% chromatic cells), and 4 (>75% chromatic cells). Scores for each pathology specimen were calculated and compared between the tissues using Pearson correlation. Here, *p* < 0.05 will be considered as statistically significant. 

### 2.3. Mouse Model of ID8 Metastatic Ovarian Cancer

All experimental procedures were performed in accordance with relevant guidelines and regulations. All experiments were approved by the Institutional Animal Committee (IACUC) at BIDMC. Animals with conditional macrophage deletion of HO-1 (*LysM-Cre:Hmox1^fl/fl^*) and control mice (*Hmox1^fl/fl^*) were generated as previously described [23]. *Hmox1^flfl^* or *LysM-Cre:Hmox1^flfl^* mice were injected with 1 × 10^6^ ID8 mouse ovarian cancer cells to their peritoneal cavity. The tumors were harvested 2 months later and evaluated by IHC. 

### 2.4. Real-Time PCR 

Total RNA was isolated from PM and PBMC using RNeasy Plus Mini Kits (QIAGEN, Valencia, CA, USA), and cDNA was synthesized with HiFiScript cDNA Synthesis Kit (CWBIO, Beijing, China) based on manufacturer instructions. Furthermore, qPCR was performed as previously described [22]. Primers were purchased from Thermo Fisher Scientific. The following oligonucleotides were used for: 

β-actin: Forward 5′-CGCGAGAAGATGACCCAGATC-3′; Reverse 5′-TCACCGGAGTCCATCACGA-3′; IL-18: Forward 5′-ATCGCTTCCTCTCGCAACAA-3′; Reverse 5′-CTTCTACTGGTTCAGCAGCCATCT-3′; IL-16: Forward 5′-ATGCCCGACCTCAACTCCTCCACT-3′; Reverse 5′-GCCACCCAGCTGCAAGATTTC -3′; Hmox1: Forward 5′ TGTGGTACAGGGAGGCCATCACC-3′; Reverse 5′CAGGATTTGTCAGAGGCCCTGAAGG-3′; IL-1β: Forward 5′-AGCTACGAATCTCCGACCAC-3′; Reverse 5′-CGTTATCCCATGTGTCGAAGAA-3′; IL-10: Forward 5′-ACGGCGCTGTCATCGATT-3′; Reverse 5′-GGCATTCTTCACCTGCTCCA-3′ and INFγ: Forward 5′- TCGGTAACTGACTTGAATGTCCA-3′; Reverse 5′- TCGCTTCCCTGTTTTAGCTGC-3′ primers. 

The following program was applied: 95 **°**C for 10 min, 95 **°**C for 15 s, 58 **°**C for 55 s, 72 **°**C for 55 s, 95 **°**C for 15 s, 60 **°**C for 1 min, and 95 **°**C for 15 s (steps #2 to #4 repeated for 40 cycles). StepOne software version 2.3 (Applied Biosystems, MA, USA) was used to calculate relative changes in mRNA levels that were normalized to the β-actin levels.

### 2.5. Cell Culture and Treatments 

Ovarian cancer OVAR3, OVAR5, ID8 and HEY cells were a gift from Dr. Dipak Panigrahy (BIDMC, Boston, MA, USA), where OVAR3 and OVAR5 were maintained in RPMI medium (Life Technologies) supplemented with 10% fetal bovine serum (Atlanta Biologicals), whereas ID8 and HEY were maintained in DMEM medium (Life Technologies) supplemented with 10% fetal bovine serum (Atlanta Biologicals) [11]. OVAR3 and OVAR5 cells were maintained in a 37 °C humidified incubator with 5% CO_2_ and 21% O_2_, while ID8 and HEY cells were maintained in 37 °C humidified incubator with 10% CO_2_ and 21% O_2_. 

### 2.6. Reagents

Hemin (referred to as heme, Sigma-Aldrich, St. Louis, MO, USA) was prepared by dissolving powder in 0.1N NaOH and then titrated with 0.1N HCl to biological pH 7.4, followed by adjustment to 10 mM concentration with 0.9% saline. Heme stock was then aliquoted and frozen at −80 °C until use; each aliquot was thawed only once. Heme-utilizing experiments were carried out in the dark at various concentration of 1–50 µM. 

### 2.7. Soft Agar Colony Assay

Here, 1 × 10^4^ ovarian cancer cells were suspended in 0.35% biotechnology-grade agarose (Bio-Rad, Hercules, CA, USA) in RPMI supplemented with 10% FBS and plated before solidifying on a solid 0.5% agarose with RPMI supplanted with 10% FBS. Medium was replaced every third day. Colonies were maintained for 2–3 weeks in a 37 °C humidified incubator, after which they were stained with methylene blue (Sigma-Aldrich, St. Louis, MO, USA) and counted.

### 2.8. Benzidine Staining

Ovarian cancer cells were seeded on cover slips in a 6-well plate and treated with various concentrations of heme (1–50 µM). O-Dianisidine stock solution was prepared (o-Dianisidine powder, sterile water, glacial acetic acid) and stored at 4 °C in foil to prevent photoreaction. A working solution was prepared by diluting the stock solution with 30% stock H_2_O_2_. Before 1 mL of the working solution was added to each well, each well was washed with PBS. Once the working solution was added, the cells were incubated for 10 min on the cover slips, and then washed 1–2 more times with PBS. The cells were then fixed in 2% PFA for 10 min, followed by 3 more washes with PBS. Hematoxylin was added to the cells for 20 s, and the cells were then washed with water 3–4 times. Ethanol (100%) was then added to the cells for 5 min and left to be air dried. The cover slips were then mounted on the slides with gelvatol mounting medium.

### 2.9. Immunoblotting

Proteins were harvested in lysis buffer (25 mM Tris-HCl, 150 mM NaCl, 1% NP-40, 100 mM NaF, 1 Complete Mini Protease Inhibitor Cocktail Tablet (Sigma-Aldrich, St. Louis, MO, USA). After sonication, lysates were centrifuged at 15,000 × g at 4 °C for 20 min. Protein concentrations were measured using the BCA Protein Kit (Thermo Fisher Scientific, Waltham, MA, USA). Then, 15–35 µg proteins were applied on 4–12% NuPAGE Bis-Tris SDS polyacrylamide gel electrophoresis in MES SDS running system (Novex, Life Technologies/Thermo Fisher Scientific, Waltham, MA, USA) followed by transfer to PVDF membrane (BioRad, Hercules, CA, USA). Following transfer, membranes were blocked in 5% nonfat milk for one hour. The following antibodies were applied rotating overnight at 4 °C: HO-1 (Enzo Life Sciences, Farmingdale, NY, USA), HO-1 rabbit monoclonal (Abcam, Cambridge, MA, USA), HO-2 (Abcam, Cambridge, MA, USA), Phospho (Ser139)-H2AX (γH2AX) (Cell Signaling, Danvers, MA, USA), cyclin B1 (Santa Cruz Biotechnology, Santa Cruz, CA, USA), Phospho (Ser1981)-ATM (Cell Signaling Technology, Danvers, MA, USA), c-myc (Santa Cruz Biotechnology), and Hemopexin (Abcam, Cambridge, MA, USA). β-Actin (Sigma-Aldrich, St. Louis, MO, USA) was used for total lysates while lamin A/C (Cell Signaling Technologies, Danvers, MA, USA) and GAPDH (Cell Signaling Technologies, Danvers, MA, USA) were used for nuclear and cytoplasmic loading controls, respectively. The following day, after brief washing with Tris-buffered saline, membranes were incubated with HRP conjugated secondary antibodies (Cell Signaling Technologies, Danvers, MA, USA), followed by chemiluminescent (ECL, Thermo Fisher Scientific, Waltham, MA, USA) detection on the ChemiDoc Imaging System (BioRad, Hercules, CA, USA). 

### 2.10. Geo Profiles

Here, mRNA expression *Geo Profiles* from 10 ovarian endometriosis and 10 matched normal endometrium biopsies from the same patient were used [24]. Geo profiles using Affymetrix Human Genome U133 Plus 2.0 gene array included tissue from 10 patients with ovarian endometriosis and 10 matched control endometrium from the same patients (follicular phase = 2, luteal phase = 8), who had not received hormone therapy before surgery [24].

### 2.11. Statistics

All data are presented as mean ± standard deviation unless otherwise indicated. Statistical analysis was performed using Student’s t-test or one-way analysis of variance (ANOVA) followed by the post-hoc Tukey test using Prism 5.0 (Graphpad Software, San Diego, CA, USA). Differences between groups were rated significant at values of *p* < 0.05.

## 3. Results

### 3.1. Heme-Associated Scavenging Proteins Are Elevated in Endometriosis

**Figure 1:**
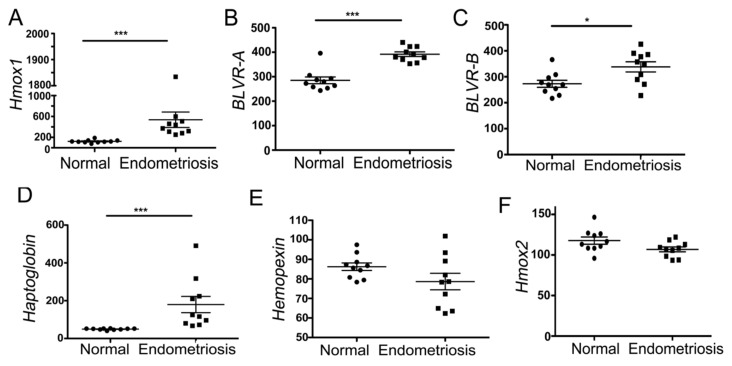
HO-1 and heme-associated protein levels in ovarian endometriosis and normal endometrium (**A**–**F**). Here, mRNA expression *Geo Profiles* from 10 ovarian endometriosis and 10 matched normal endometrium biopsies from the same patient were used [24]. Mean values ± SD are shown. * *p* < 0.05. *** *p* < 0.001.

We analyzed the RNA microarray expression profiles of ovarian endometriosis (*n* = 0) and normal endometrium (*n* = 10) from the same patients previously deposited in the *Geo Profiles* database [24]. Compared to normal endometrium, endometriosis samples showed elevated levels of heme-degradation-pathway-associated genes: *Hmox1* (encoding HO-1), *BLVR-A* and *BLVR-B*, and *hemopexin (Hx)* as well as *haptoglobin (Hp*), a hemoglobin scavenger (Figure 1A–D). Endometriosis showed similar or slightly lower levels of *Hmox2* (encoding HO-2) and Hx (Figure 1E,F). 

### 3.2. High Expression of CD45^+^ and HO-1^+^ Cells Infiltration in Endometrioma and Associated EAOC 

**Figure 2:**
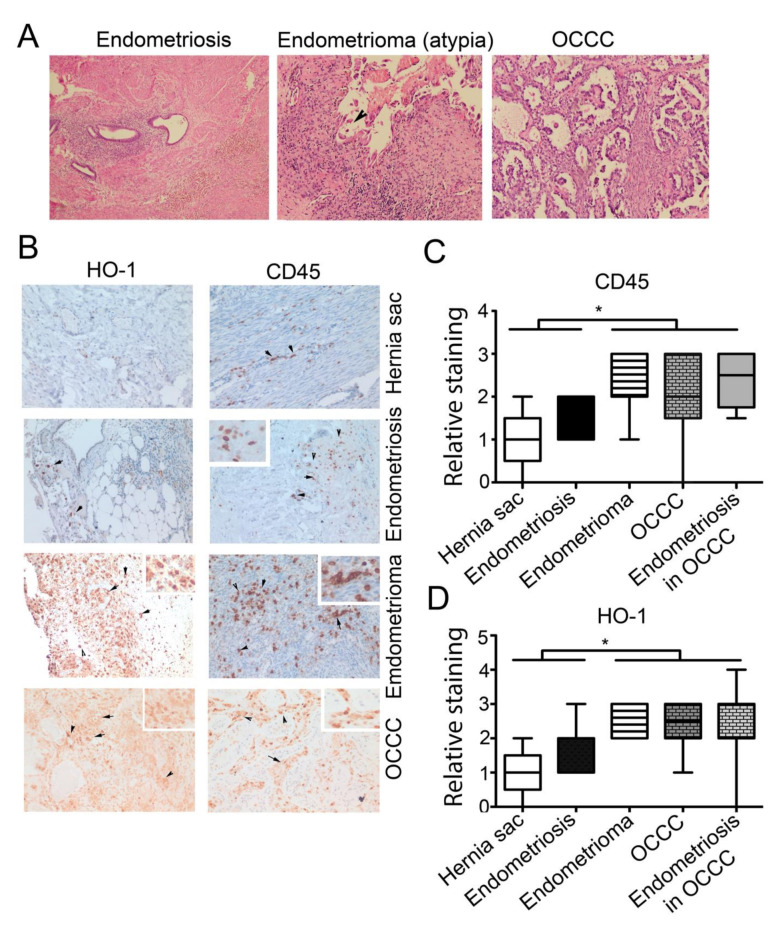
Expression of CD45, HO-1 and Hx in patients with EAOC. (**A**) Representative histology from the following lesions: peritoneal endometriosis, ovarian endometrioma, EAOC clear cell carcinoma. Magnification 200×. (**B**) Representative lesions and control (hernia sac) immunostained for HO-1 and CD45 highlight stromal macrophages. Magnification 200× with 400× inserts. (**C**,**D**) The graphs showing the relative intensities of stained cells (macrophages), which are higher in cancer tissue and endometriosis lesions in women with cancer, than in endometriosis alone or controls. Mean values +/− SEM are shown. * *p* < 0.05.

To assess the pattern of the expression of HO-1 in the endometriosis and OCCC, we employed archived biopsies from patients with clear cell EAOC (*n* = 17), endometrioma, a cystic endometriosis arising in the ovary (*n* = 5), endometriosis (*n* = 6) or control tissue from hernia sacs (*n* = 5). Clinical characteristics of EAOC patients are shown in Supplementary Table S1. The vast majority of EAOC patients were low-stage and treated with platinum-based chemotherapy without radiation. The representative H&E staining is shown in Figure 2A. We measured the levels of HO-1 and CD45, a marker of infiltrating leukocytes, in the cancer tissue and in the adjacent endometriosis as well as endometrioma, endometriosis and normal hernia sac tissues, using IHC staining (Figure 2B). HO-1 (nuclear and cytoplasmic staining) was primarily detected in macrophages in the stroma of endometriotic lesions (Figure 2B). HO-1 staining was highest in cases with high CD45^+^ cell infiltration. No HO-1 staining of epithelial cells in endometriosis was seen, but moderately intense staining (nuclear and cytoplasmic) was seen in cancer cells of the adjacent EAOC (Figure 2B). We observed significantly higher expression of HO-1 in endometrioma and OCCC, correlating well with a high level of CD45^+^ cells (Figure 2B–D). Further, there was a positive correlation between the number of CD45^+^ and HO-1^+^ cells in the endometriosis regions associated with OCCC (*p* = 0.008; *r*^2^ = 0.636), indicating their co-expression (Supplementary Table S2). There was high variation in the immune cell infiltrate between EAOC cases; however, the intensity of staining was significantly higher in endometrioma, in cancer and endometriosis adjacent to EAOC compared to biopsies from hernia sac and patient with endometriosis (Figure 2C). A high uniform expression of Hx was observed in endometriosis associated with EAOC as well as in regions of cancer stroma (Supplementary Figure S1A–B), but was not significantly different between them (Supplementary Figure S1A–B). Strong expression of Hx was detected in infiltrating stroma cells (Supplementary Figure S1A–B).

### 3.3. Peritoneal Macrophages from Women with Endometriosis Express High Levels of HO-1 and IL-10, Suggesting a Stromal M2-Like Phenotype 

**Figure 3:**
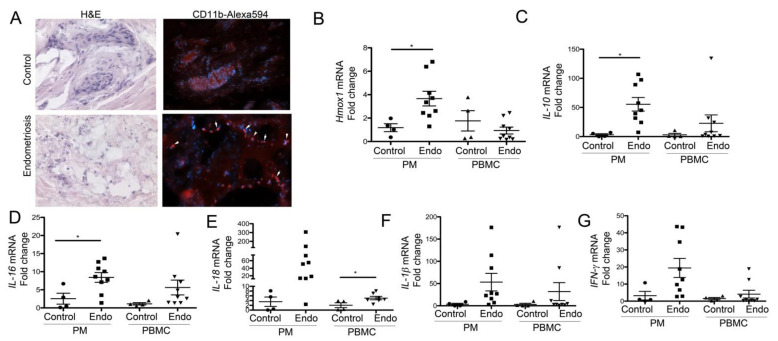
Immune phenotype of endometriosis, peritoneal macrophage and peripheral blood monocytes. (**A**) Histological analysis of endometriotic and control peritoneal biopsies. Immunofluorescent staining with antibodies against CD11b is shown against H&E staining in the endometriosis and control peritoneal biopsies. Magnification 400x. (B–**G**) mRNA levels of HO-1 IL-10, IL-16, IL-18, IL-1β and IFNγ in patients with endometriosis (ENDO, *n* = 9) and control subjects (*n* = 4). * *p* < 0.05.

We collected peritoneal pellets (macrophages), biopsies (endometriosis or normal tissue) and PBMC from *n* = 4 control (surgery due to uterine fibroid removal, genetic susceptibility to ovarian cancer/neoplasm, lesion on cervix) and *n* = 9 endometriosis patients. The endometriosis lesions were strongly infiltrated by CD11b^+^ positive cells (Figure 3A). Importantly, we detected elevated levels of HO-1 in peritoneal pellets from patients with endometriosis (Figure 3B). In contrast, no increase in HO-1 was seen in the PBMC (Figure 3B). IL-10 levels were significantly higher in the PM in endometriosis patients, and a similar trend was seen in PBMC (Figure 3C). Moreover, there was a positive correlation between the HO-1 and IL-10 levels in the PM (*p* = 0.002, *r*^2^ = 0.776, Pearson), confirming a previously reported direct interplay between HO-1 and IL-10 [25]. The strong induction of HO-1 and IL-10 in peritoneum in endometriosis patients indicate M2-like skewing of macrophages. We found a significant induction of IL-16 in PM (Figure 3D) and IL-18 in PBMC (Figure 3E) isolated from patients with endometriosis. The trends of increased levels of IL-16, IL-18, IL-1β and IFNγ were seen both in PM and PBMC in endometriosis patients (Figure 3F,G).

### 3.4. Heme Modulates the Growth of Ovarian Cancer Colonies in Soft Agar

**Figure 4:**
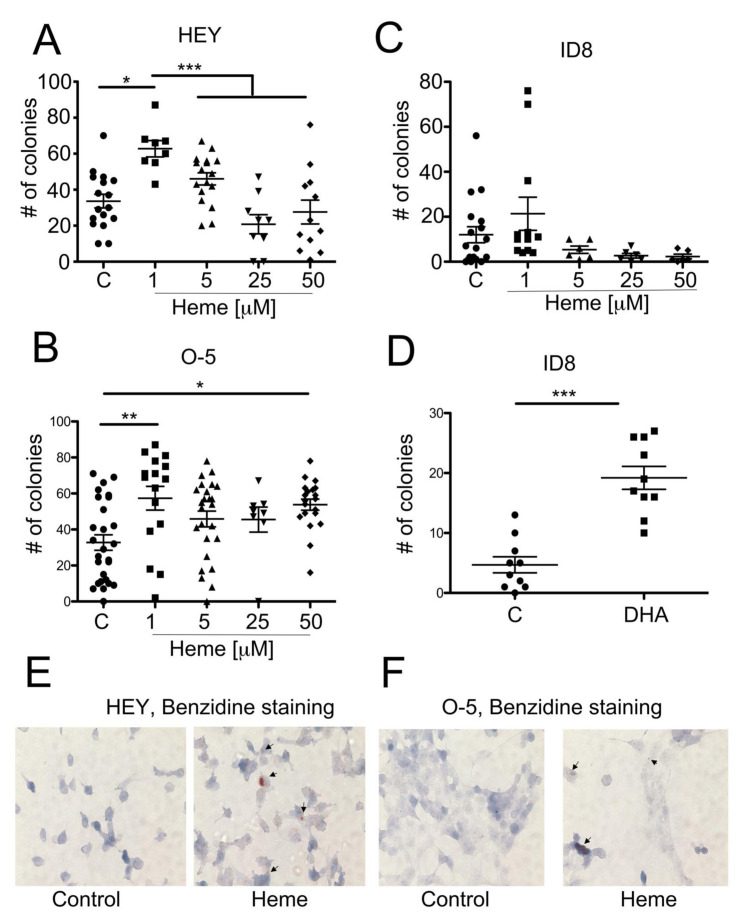
Heme accumulation in cancer cells is associated with anchorage-independent growth. (**A**–**C**) Anchorage-independent growth in soft agar of HEY and OVAR-5 (O-5) and ID-8 ovarian cancer cells treated with heme (1–50 µM) was assessed at 3 weeks. * *p* < 0.05, ** *p* < 0.01, *** *p* < 0.001. Data are representative for *n* = 4–5 experiments in duplicates or triplicates. (**D**) ID8 cells were treated with DHA, an inhibitor of ALA synthetase and colony growth was evaluated at 3 weeks. *** *p* < 0.001. Data are representative for *n* = 6. (**E**,**F**) The heme levels were measured by the benzidine staining in HEY and OVAR-5 cells treated with heme for 24 h. Magnification 400x.

Our observations from the clinical material prompted us to investigate the role of heme in in vitro cultures of ovarian cancer cell lines. Based on the *Geo profiles* data comparing ovarian clear-cell-like cancer cell lines with serous ovarian cancer cell lines, there was no difference in the baseline levels of expression of HO-1, Hx or Hp between the cell lines (Supplementary Figure S2A–C). We chose to assess the role of labile heme in cultured HEY, ID8 and OVCAR-5 ovarian cancer cell lines (Figure 4). OVCAR-5 (O-5) is a human epithelial carcinoma cell line of the ovary, established from the ascitic fluid of a patient with progressive ovarian adenocarcinoma without prior cytotoxic treatment. The HEY human ovarian carcinoma cell line was derived from a human ovarian cancer xenograft (HX-62) originally grown from a peritoneal deposit of a patient with moderately differentiated papillary cystadenocarcinoma of the ovary. Finally, the ID8 ovarian cancer cell line was derived from mouse ovarian surface epithelial cells that were transformed by serial passage in vitro. We demonstrated the higher number of colonies in soft agar in the presence of low doses of heme (1 µM) in the O-5 and HEY cell lines (Figure 4A,B), without significant changes in the growth of the ID8 cell line (Figure 4C). Interestingly, heme at higher levels (5–50 µM) did not influence the cancer cell growth of HEY cells (Figure 4A) and only slightly increased the number of colonies in the O-5 cell line (Figure 4B). There was no significant change in the colony number of ID8 cells, yet the there was a trend towards lower numbers of colonies at higher doses of heme (Figure 4C). To assess the role of endogenous heme synthesis in ID8 colonies growth, we treated ID8 cells during culture in soft agar with DHA, an inhibitor of ALA-synthase-1 and heme synthesis (Figure 4D). Interestingly, we found higher colony number in cells treated with DHA (Figure 4D), suggesting that a decrease in endogenous levels of heme may promote cancer cell growth similarly to low levels of exogenous heme. We have confirmed the uptake and accumulation of heme in HEY and O-5 cancer cells upon treatment with exogenous heme using benzidine staining (Figure 4E,F).

### 3.5. Heme Induces HO-1 in Ovarian Cancer Cell Lines

**Figure 5:**
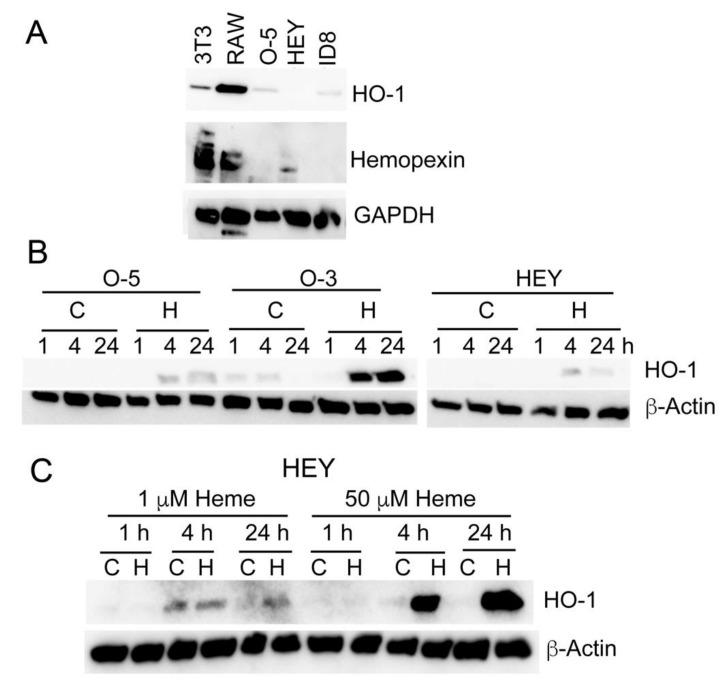
Heme induces HO-1, Hx and DNA damage in ovarian cell lines. (**A**) Immunoblot analysis with antibody against HO-1 and Hx in NIH3T3 fibroblasts, RAW macrophage cell line and ovarian cell lines. (**B)** HO-1 and HO-2 were determined by immunoblotting lysates of HEY and OVAR-5 (OV-5), OVAR-3 (O-3) ovarian cancer cells treated with heme (H, 50 µM) or control (**C**) for 1–24h. Treatment of HEY cells with heme at 1 and 50 µM. Immunoblot analysis of HO-1 levels is shown at 1, 4, and 24 h. Please see the whole western blots in File S1.

Low levels of baseline HO-1 and Hx expression were detected in ovarian cancer cell lines compared to stroma cells, NIH3T3 fibroblasts and RAW267.4 macrophage cell lines (Figure 5A). We found that HO-1 was induced as early as 4 h in response to high doses of heme (50 µM) (Figure 5B). Interestingly, the lower doses of heme (1 µΜ) that induced cancer cell growth in soft agar, had a very little effect on HO-1 induction in HEY cells (Figure 5C).

### 3.6. Lack of HO-1 in Macrophages Results in Attenuated Growth of ID8 Tumors In Vivo 

**Figure 6:**
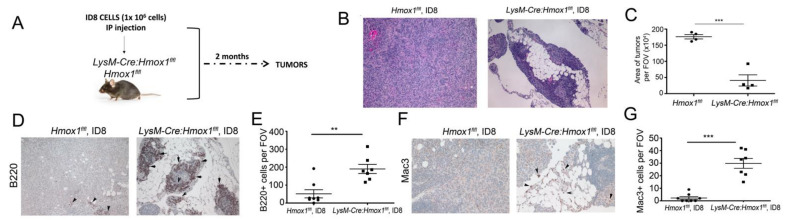
Lack of HO-1 in macrophages (*LysM-Cre:Hmox1^flfl^* mice) suppresses growth of ID8 tumors in metastatic model of ovarian cancer. (**A**) Scheme showing the experimental design. (**B**) Representative H&E staining of ID8 tumors growing in the peritoneum of *Hmox1* or *LysM-Cre:Hmox1^flfl^* mice and harvested at 8 weeks after inoculation. Magnification 200x. (**C**) Quantification of the size of the tumors based on the histology. *N* = 4-5 mice/group. *** *p* < 0.001. (**D**–**G**) IHC analyses of ID8 tumors examined as in Figure 1 and stained with antibodies against B220 (**D**,**E**) or Mac3 (**F**,**G**). Magnification 200x. The representative sections are shown in (**D**,**F**), and quantification is shown in (**E**,**G**). ** *p* < 0.01, *** *p* < 0.001.

We established the metastatic syngeneic model of ovarian cancer by inoculating ID8 mouse ovarian cancer cells into the peritoneum of *Hmox1^flfl^* or *LysM-Cre:Hmox1^flfl^* mice (Figure 6A). By immunohistochemical (IHC) analyses, we noted smaller tumors spread in the peritoneal cavity in the *LysM-Cre:Hmox1^flfl^* mice, with large numbers of leukocytes infiltrating into the tumor niche compared to *Hmox1^flfl^* mice at 8 weeks after inoculation (Figure 6B,C). Since the deletion of HO-1 in macrophages leads to a significant increase in the number of immune cells infiltrating into the tumors (Figure 6B), we assessed the presence of B220+ (B cells) and Mac3/LAMP-2+ (macrophages) in those tumors (Figure 6D,E). We found significantly higher numbers of B220+ and Mac3/LAMP-2+ in *LysM-Cre:Hmox1^flfl^* mice correlating with smaller tumors (Figure 6D,E), suggesting a pronounced immune responses in the tumor microenvironment.

## 4. Discussion

Endometriosis affects between 10–15% of women in the US during their reproductive years [26], with a 0.7% to 2% lifetime risk of EAOC [27]. Cancers are of clear cell and endometrioid histology and arise by malignant transformation of either benign or atypical endometriotic lesions [28,29], in part due to the microenvironment created by trapped blood and cellular debris created during menstrual cycling in the abnormal ectopic location [6]. In this study, we demonstrated that labile heme and HO-1 are critical components of endometriotic niches that may play important roles in endometriosis progression to clear cell cancer. 

We showed that HO-1 levels are significantly increased in endometriotic lesions, specifically, within peritoneal macrophages (PM) isolated from endometriotic patients. This appears to be driven locally, since this was not evident in PBMC from the same patients. Interestingly, PMs from patients with endometriosis also exhibited elevated levels of IL-10 and IL-16. Similar trends in expression were observed for IL-1β, Il-18 and IFNγ; however, due to small number of cases, they did not reach significance. These cytokines have previously been associated with malignant progression of endometriosis to EAOC [30,31,32]. Anti-inflammatory mediators such as IL-10 are essential for M2-like macrophage skewing and HO-1 induction, which is prominent in endometriotic lesions. Interestingly, unlike HO-1 expression, many of these changes in cytokines levels were not confined to cells isolated from the peritoneum but were also detected in the mRNA isolated from endometriotic patient PMBCs. This data may indicate that endometriosis likely represents a systemic inflammatory disease.

By IHC, we found that HO-1 is highly expressed in the infiltrating immune cells in endometrioma (atypical lesions and strongly associated with OCCC) and carcinoma, with a few positive cells in endometriosis compared to hernia sac control tissues. Both HO-1 and Hx were strongly expressed in macrophages associated with CD45 positive immune cell infiltrates; however, Hx was also strongly stained in stromal, epithelial and cancer cells. We have previously reported that Hx is a tumor suppressor in mouse models of prostate cancer and high levels of Hx in the stroma in human biopsies correlated with better outcomes in prostate-cancer patients [10]. High levels of Hx in the endometriosis stroma may be associated with large loads of heme to scavenge. The level of HO-1 and Hx maintains the balance of free heme in the environment and possibly the skewing of macrophages [33]. We showed that HO-1 expression and the density of stromal macrophages is higher in lesions adjacent to cancer. This raises the possibility that a tumor-like stroma is a prerequisite for progression to cancer. This observation is consistent with the epidemiology data showing that endometriosis-associated cancers occur after many years of persistent endometriosis.

Our studies demonstrated deletion of HO-1 in myeloid cells in the ovarian tumor niche resulted in smaller tumors. Thus, macrophage-expressed HO-1 promotes tumor growth in part by removing the high heme levels in the tumor microenvironment, which would otherwise be available to cancer cells. This is supported by our in vitro data, which shows that high levels of heme suppress ovarian cancer cell growth, while low levels of heme promote growth of the cell lines.

Smaller tumors in HO-1 conditional knockout mice were associated with infiltration of Mac3^+^ leukocytes and B220^+^ B cells. B cells have been associated with both pro-tumoral and anti-tumoral responses [34] and their entering into TME depends on the presence of specific chemokines. CXCL12 (SDF-1a) is not only a B cell activator [35] but also a target of HO-1 [36] and is upregulated in HO-1-deficient macrophages (based on our RNA-seq data), which may at least partially explain the increase in B cells detected here. In metastatic ovarian cancer, B cells show cytolytic responses against tumors. Heme has also been shown to skew B cells towards producing the IgM rather than the IgG phenotype [37]. Future study will aim to assess the role of Hx and heme in the TME in ovarian cancer models. 

In our in vitro experimentation, we demonstrated that heme accumulation within ovarian cancer cells was similar to our previous observations in prostate cancer [10]. However, unlike in prostate cancer cells, in vitro treatment of ovarian cancer cells with heme, induced anchorage-independent growth only at a low (1 µM) concentration, but growth remained unchanged or lower at heme concentrations >5 µM. This high-dose effect was associated with induction of HO-1. Levels of HO-1 were not highly induced with 1 µM heme. Macrophage-expressed HO-1 may promote tumor growth by removing the high heme levels in the tumor microenvironment, which would be otherwise available to cancer cells to support their growth. These data suggest that perturbation of heme metabolism within the endometriotic niche and in cancer cells themselves may be an important factor that influences tumor initiation and growth. Others have speculated on combining chemotherapy with an HO-1 inhibitor in other tumors such as pancreatic cancer, in order to overcome stroma-driven chemotherapy resistance [38].

The concentration of heme in endometriosis depends on the age of the lesions (older lesions typically scar over rather than cycle), Hx levels and heme metabolism in macrophages, and the number of macrophages in the lesions. We showed that HO-1 and Hx are highly associated with endometriosis and clear cell EAOC. A role of heme in supporting the progression of endometriosis to malignancy could explain the fact that EAOC generally occurs in women with long-standing endometriosis, and in endometriotic cysts (high HO-1, low heme) rather than active peritoneal endometriosis (high heme, low HO-1). Pre-menopausal women would be expected to have endometriosis with high levels of heme, leading to the suppression of neoplastic growth. However, over time, there would be recruitment of HO-1-expressing macrophages to decrease heme concentrations to a level promoting tumor growth. HO-1 expression favors type 2 macrophages, promoting fibrosis in mature endometriosis lesions, and a tumor-supportive microenvironment. As women enter menopause, heme levels decrease due to a loss of cyclic bleeding. All these factors create conditions favoring neoplastic transformation, which occurs due to genetic and epi-genetic properties intrinsic to the epithelial cells. Oxidative stress in an endometriotic cyst may also be a driver of cancer. Our observation of the nuclear staining of HO-1, associated previously with lower enzymatic activity, in OCCC, may also reflect a response to oxidative stress and progression of cancer. Nrf2, a cellular sensor of oxidative stress and a regulator of HO-1, is known to be elevated in EAOC [39]. 

## 5. Conclusions

Our study suggests that fluctuation in heme levels and HO-1 expression may explain the epidemiology of EAOC in post-menopausal women and in endometriotic cysts of the ovary. 

## Data Availability

The data presented in this study are available in this article (and Supplementary Materials).

## Supplementary Materials

The following supporting information can be downloaded at: https://www.mdpi.com/article/10.3390/cancers14092242/s1, Figure S1: The expression of Hx in the cancer and adjacent endometriosis in OCCC patients as in Figure 2. The representative sections are shown in A. Quantification is shown in B.; Figure S2: Geo Profile comparison of the relative expression of Hmox1, Hx and Hp in the ovarian clear cell-like cancer (OCC) or adenocarcinoma of ovary cell lines; Table S1: Clinical characteristic of patients with EAOC used for tissue immunohistochemistry; Table S2: Correlation (r2 and p values) between the staining intensities in patients with EAOC. *n* = 17 patients. S-stroma; T-tumor; Hx-hemopexin, HO-1-heme oxygenase-1. Supplementary File S1 (original blots).

## References

[1] Vercellini P., Vigano P., Somigliana E., Fedele L. (2014). Endometriosis: Pathogenesis and treatment. Nat. Rev. Endocrinol..

[2] Anglesio M.S., Papadopoulos N., Ayhan A., Nazeran T.M., Noe M., Horlings H.M., Lum A., Jones S., Senz J., Seckin T. (2017). Cancer-Associated Mutations in Endometriosis without Cancer. N. Engl. J. Med..

[3] Lac V., Nazeran T.M., Tessier-Cloutier B., Aguirre-Hernandez R., Albert A., Lum A., Khattra J., Praetorius T., Mason M., Chiu D. (2019). Oncogenic mutations in histologically normal endometrium: The new normal?. J. Pathol..

[4] Munksgaard P.S., Blaakaer J. (2012). The association between endometriosis and ovarian cancer: A review of histological, genetic and molecular alterations. Gynecol. Oncol..

[5] Donnez J., Binda M.M., Donnez O., Dolmans M.M. (2016). Oxidative stress in the pelvic cavity and its role in the pathogenesis of endometriosis. Fertil. Steril..

[6] Kobayashi H. (2016). Potential scenarios leading to ovarian cancer arising from endometriosis. Redox Rep..

[7] Van Langendonckt A., Casanas-Roux F., Donnez J. (2002). Iron overload in the peritoneal cavity of women with pelvic endometriosis. Fertil. Steril..

[8] Gozzelino R., Jeney V., Soares M.P. (2010). Mechanisms of cell protection by heme oxygenase-1. Annu. Rev. Pharmacol. Toxicol..

[9] Canesin G., Hejazi S.M., Swanson K.D., Wegiel B. (2020). Heme-Derived Metabolic Signals Dictate Immune Responses. Front. Immunol..

[10] Canesin G., Di Ruscio A., Li M., Ummarino S., Hedblom A., Choudhury R., Krzyzanowska A., Csizmadia E., Palominos M., Stiehm A. (2020). Scavenging of Labile Heme by Hemopexin Is a Key Checkpoint in Cancer Growth and Metastases. Cell Rep..

[11] Wegiel B., Gallo D., Csizmadia E., Harris C., Belcher J., Vercellotti G.M., Penacho N., Seth P., Sukhatme V., Ahmed A. (2013). Carbon monoxide expedites metabolic exhaustion to inhibit tumor growth. Cancer Res..

[12] Sacca P., Meiss R., Casas G., Mazza O., Calvo J.C., Navone N., Vazquez E. (2007). Nuclear translocation of haeme oxygenase-1 is associated to prostate cancer. Br. J. Cancer.

[13] Tibullo D., Barbagallo I., Giallongo C., La Cava P., Parrinello N., Vanella L., Stagno F., Palumbo G.A., Li Volti G., Di Raimondo F. (2013). Nuclear translocation of heme oxygenase-1 confers resistance to imatinib in chronic myeloid leukemia cells. Curr. Pharm. Des..

[14] Hsu F.F., Yeh C.T., Sun Y.J., Chiang M.T., Lan W.M., Li F.A., Lee W.H., Chau L.Y. (2015). Signal peptide peptidase-mediated nuclear localization of heme oxygenase-1 promotes cancer cell proliferation and invasion independent of its enzymatic activity. Oncogene.

[15] Lin Q., Weis S., Yang G., Weng Y.H., Helston R., Rish K., Smith A., Bordner J., Polte T., Gaunitz F. (2007). Heme oxygenase-1 protein localizes to the nucleus and activates transcription factors important in oxidative stress. J. Biol. Chem..

[16] Kobayashi H., Sumimoto K., Moniwa N., Imai M., Takakura K., Kuromaki T., Morioka E., Arisawa K., Terao T. (2007). Risk of developing ovarian cancer among women with ovarian endometrioma: A cohort study in Shizuoka, Japan. Int. J. Gynecol. Cancer.

[17] Capobianco A., Rovere-Querini P. (2013). Endometriosis, a disease of the macrophage. Front. Immunol..

[18] Wolfler M.M., Meinhold-Heerlein I.M., Henkel C., Rath W., Neulen J., Maass N., Brautigam K. (2013). Reduced hemopexin levels in peritoneal fluid of patients with endometriosis. Fertil. Steril..

[19] Tai C.S., Lin Y.R., Teng T.H., Lin P.Y., Tu S.J., Chou C.H., Huang Y.R., Huang W.C., Weng S.L., Huang H.D. (2017). Haptoglobin expression correlates with tumor differentiation and five-year overall survival rate in hepatocellular carcinoma. PLoS ONE.

[20] Kobayashi S., Nouso K., Kinugasa H., Takeuchi Y., Tomoda T., Miyahara K., Hagihara H., Kuwaki K., Onishi H., Nakamura S. (2012). Clinical utility of serum fucosylated hemopexin in Japanese patients with hepatocellular carcinoma. Hepatol. Res..

[21] Chang Y.K., Lai Y.H., Chu Y., Lee M.C., Huang C.Y., Wu S. (2016). Haptoglobin is a serological biomarker for adenocarcinoma lung cancer by using the ProteomeLab PF2D combined with mass spectrometry. Am. J. Cancer Res..

[22] Bisht K., Canesin G., Cheytan T., Li M., Nemeth Z., Csizmadia E., Woodruff T.M., Stec D.E., Bulmer A.C., Otterbein L.E. (2019). Deletion of Biliverdin Reductase A in Myeloid Cells Promotes Chemokine Expression and Chemotaxis in Part via a Complement C5a--C5aR1 Pathway. J. Immunol..

[23] Hedblom A., Hejazi S.M., Canesin G., Choudhury R., Hanafy K.A., Csizmadia E., Persson J.L., Wegiel B. (2019). Heme detoxification by heme oxygenase-1 reinstates proliferative and immune balances upon genotoxic tissue injury. Cell Death Dis..

[24] Hever A., Roth R.B., Hevezi P., Marin M.E., Acosta J.A., Acosta H., Rojas J., Herrera R., Grigoriadis D., White E. (2007). Human endometriosis is associated with plasma cells and overexpression of B lymphocyte stimulator. Proc. Natl. Acad. Sci. USA.

[25] Otterbein L.E., Soares M.P., Yamashita K., Bach F.H. (2003). Heme oxygenase-1: Unleashing the protective properties of heme. Trends Immunol..

[26] Kalaitzopoulos D.R., Mitsopoulou A., Iliopoulou S.M., Daniilidis A., Samartzis E.P., Economopoulos K.P. (2020). Association between endometriosis and gynecological cancers: A critical review of the literature. Arch. Gynecol. Obstet..

[27] Grandi G., Toss A., Cortesi L., Botticelli L., Volpe A., Cagnacci A. (2015). The Association between Endometriomas and Ovarian Cancer: Preventive Effect of Inhibiting Ovulation and Menstruation during Reproductive Life. Biomed. Res. Int..

[28] Ahn S.H., Khalaj K., Young S.L., Lessey B.A., Koti M., Tayade C. (2016). Immune-inflammation gene signatures in endometriosis patients. Fertil. Steril..

[29] Jiang L., Yan Y., Liu Z., Wang Y. (2016). Inflammation and endometriosis. Front. Biosci (Landmark Ed.).

[30] Leenen S., Hermens M., de Vos van Steenwijk P.J., Bekkers R.L.M., van Esch E.M.G. (2021). Immunologic factors involved in the malignant transformation of endometriosis to endometriosis-associated ovarian carcinoma. Cancer Immunol. Immunother..

[31] Wendel J.R.H., Wang X., Hawkins S.M. (2018). The Endometriotic Tumor Microenvironment in Ovarian Cancer. Cancers.

[32] Koga K., Osuga Y., Yoshino O., Hirota Y., Yano T., Tsutsumi O., Taketani Y. (2005). Elevated interleukin-16 levels in the peritoneal fluid of women with endometriosis may be a mechanism for inflammatory reactions associated with endometriosis. Fertil. Steril..

[33] Zhang M., Nakamura K., Kageyama S., Lawal A.O., Gong K.W., Bhetraratana M., Fujii T., Sulaiman D., Hirao H., Bolisetty S. (2018). Myeloid HO-1 modulates macrophage polarization and protects against ischemia-reperfusion injury. JCI Insight.

[34] Mantovani A. (2011). B cells and macrophages in cancer: Yin and yang. Nat. Med..

[35] Nagasawa T., Kikutani H., Kishimoto T. (1994). Molecular cloning and structure of a pre-B-cell growth-stimulating factor. Proc. Natl. Acad. Sci. USA.

[36] Deshane J., Chen S., Caballero S., Grochot-Przeczek A., Was H., Li Calzi S., Lach R., Hock T.D., Chen B., Hill-Kapturczak N. (2007). Stromal cell-derived factor 1 promotes angiogenesis via a heme oxygenase 1-dependent mechanism. J. Exp. Med..

[37] Watanabe-Matsui M., Muto A., Matsui T., Itoh-Nakadai A., Nakajima O., Murayama K., Yamamoto M., Ikeda-Saito M., Igarashi K. (2011). Heme regulates B-cell differentiation, antibody class switch, and heme oxygenase-1 expression in B cells as a ligand of Bach2. Blood.

[38] Ahmad I.M., Dafferner A.J., O'Connell K.A., Mehla K., Britigan B.E., Hollingsworth M.A., Abdalla M.Y. (2021). Heme Oxygenase-1 Inhibition Potentiates the Effects of Nab-Paclitaxel-Gemcitabine and Modulates the Tumor Microenvironment in Pancreatic Ductal Adenocarcinoma. Cancers.

[39] Konstantinopoulos P.A., Spentzos D., Fountzilas E., Francoeur N., Sanisetty S., Grammatikos A.P., Hecht J.L., Cannistra S.A. (2011). Keap1 mutations and Nrf2 pathway activation in epithelial ovarian cancer. Cancer Res..

