# Supplementary material for: Labile Heme and Heme Oxygenase-1 Maintain Tumor-Permissive Niche for Endometriosis-Associated Ovarian Cancer
Source: Cancers (Basel). 2022 Apr 29;14(9):2242. doi: 10.3390/cancers14092242 (PMC9105072; doi:10.3390/cancers14092242)
Supplement: Supplementary file 1 [file cancers-14-02242-s001.zip › cancers-1641564-supplementary.pdf]

Supplementary Figures and Tables:

Table S1: Clinical characteristic of patients with EAOC used for tissue immunohistochemistry.

Patient #	Age	Stage	Chemotherapy (Y/N)	Radiation (Y/N)	Time from diagnosis to last seen at the clinic (months)
1	45	IA	Y	N	78
2	48	IC3	Y	N	102
3	41	IC1	Y	N	29
4	51	IA	Y	N	70
5	48	IIA	Y	N	204
6	50	IC3	Y	N	148
7	64	IA	Y	N	124
8	66	IIB	Y	N	74
9	49	IIB	Y	Y	92
10	53	IC1	N	N	1
11	43	IA	Y	N	46
12	82	IIB	-	-	1
13	53	IC3	Y	N	15
14	46	IC1	N	N	14
15	50	IC1	N	N	24
16	51	IIA	Y	N	9
17	45	IC1	Y	N	N/A

Table S2. Correlation (r^2 and p values) between the staining intensities in patients with EAOC. n=17 patients. S-stroma; T-tumor; Hx-hemopexin, HO-1-heme oxygenase-1.

p values	**Hx-T**	**CD45-T**	**HO-1-T**	**Hx-S**	**HO-1-S**	**Hx Endo**	**CD45 Endo**	**HO-1 Endo**	**Hx Endo-S**	**HO-1 Endo-S**	**Age**	**Chemo**	**Radiation**	**Stage**
**Hx-T**		0.957	0.924	0.011		**0.040**	0.965	0.636	0.158	0.177	0.908	0.780	0.470	0.502
**CD45-T**	0.957		0.691	0.163	0.416	0.532	0.115	0.708	0.914	0.570	0.197	0.559	0.515	0.542
**HO-1-T**	0.924	0.691		0.696	0.002	0.465	0.067	0.357	0.152	0.846	0.335	0.853	0.879	0.634
**Hx-S**	0.011	0.163	0.696		0.909	0.354	0.586	0.646	0.173	0.612	0.939	0.756	0.096	**0.037**
**HO-1-S**		0.416	0.002	0.909		0.938	0.568	0.824	0.426	0.859	0.590	0.735	0.880	0.511
**Hx Endo**	**0.040**	0.532	0.465	0.354	0.938		0.926	0.204	0.130	0.819	0.946	0.345	0.071	0.426
**CD45 Endo**	0.965	0.115	0.067	0.586	0.568	0.926		0.008	0.982	0.474	0.488	0.860	0.811	0.740
**HO-1 Endo**	0.636	0.708	0.357	0.646	0.824	0.204	**0.008**		0.259	0.097	0.856	0.147	0.443	0.908
**Hx Endo-S**	0.158	0.914	0.152	0.173	0.426	0.130	0.982	0.259		0.627	0.773	0.642		0.823
**HO-1 Endo-S**	0.177	0.570	0.846	0.612	0.859	0.819	0.474	0.097	0.627		0.009	0.691	0.550	0.107
**age**	0.908	0.197	0.335	0.939	0.590	0.946	0.488	0.856	0.773	0.009		0.581	1.000	0.144

**Chemo**	0.780	0.559	0.853	0.756	0.735	0.345	0.860	0.147	0.642	0.691	0.581		0.700	0.369
**Radiation**	0.470	0.515	0.879	0.096	0.880	0.071	0.811	0.443		0.550	1.000	0.700		0.098
**Stage**	0.502	0.542	0.634	**0.037**	0.511	0.426	0.740	0.908	0.823	0.107	0.144	0.369	0.098	

r2	**Hx-T**	**CD45-T**	**HO-1-T**	**Hx-S**	**HO-1-S**	**Hx Endo**	**CD45 Endo**	**HO-1 Endo**	**Hx Endo-S**	**HO-1 Endo-S**	**Age**	**Chemo**	**Radiation**	**Stage**
**Hx-T**		0.016	-0.028	0.679		**0.625**	-0.013	0.145	0.512	-0.494	-0.034	0.090	-0.231	-0.196
**CD45-T**	0.016		-0.117	0.411	-0.259	-0.201	0.440	-0.110	-0.042	-0.205	0.353	-0.179	-0.199	-0.171
**HO-1-T**	-0.028	-0.117		0.126	0.771	-0.247	-0.522	-0.279	0.556	0.076	-0.278	-0.060	-0.049	-0.140
**Hx-S**	0.679	0.411	0.126		0.042	0.310	0.175	0.148	0.498	-0.213	-0.023	-0.106	-0.527	**-0.582**
**HO-1-S**		-0.259	0.771	0.042		-0.031	-0.194	0.076	0.361	-0.075	-0.173	-0.116	0.052	0.211
**Hx Endo**	**0.625**	-0.201	-0.247	0.310	-0.031		0.029	0.377	0.512	-0.097	-0.020	0.299	-0.538	0.231
**CD45 Endo**	-0.013	0.440	-0.522	0.175	-0.194	0.029		0.636	-0.009	0.257	0.187	0.052	-0.070	-0.090

**HO-1 Endo**	0.145	-0.110	-0.279	0.148	0.076	0.377	**0.636**		0.421	0.553	0.049	0.408	-0.223	-0.032
**Hx Endo-S**	0.512	-0.042	0.556	0.498	0.361	0.512	-0.009	0.421		-0.254	0.105	0.196		0.082
**HO-1 Endo-S**	-0.494	-0.205	0.076	-0.213	-0.075	-0.097	0.257	0.553	-0.254		-0.772	0.168	-0.250	-0.540
**age**	-0.034	0.353	-0.278	-0.023	-0.173	-0.020	0.187	0.049	0.105	-0.772		-0.155	0.000	0.370
**Chemo**	0.090	-0.179	-0.060	-0.106	-0.116	0.299	0.052	0.408	0.196	0.168	-0.155		0.113	0.250
**Radiation**	-0.231	-0.199	-0.049	-0.527	0.052	-0.538	-0.070	-0.223		-0.250	0.000	0.113		0.443
**Stage**	-0.196	-0.171	-0.140	**-0.582**	0.211	0.231	-0.090	-0.032	0.082	-0.540	0.370	0.250	0.443	

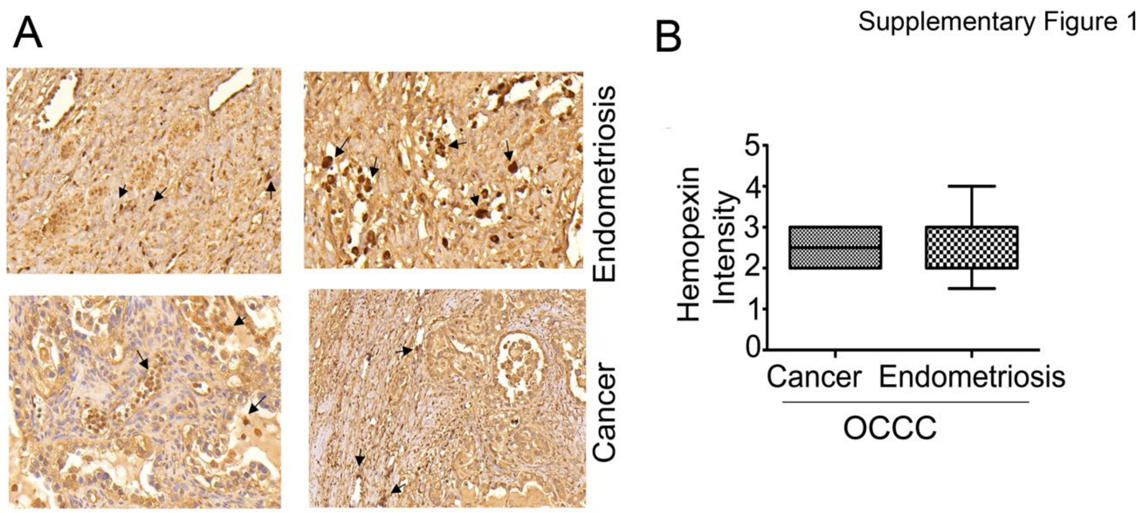

Figure S1: The expression of Hx in the cancer and adjacent endometriosis in OCCC patients as in Figure 2. The representative sections are shown in A. Quantification is shown in B.

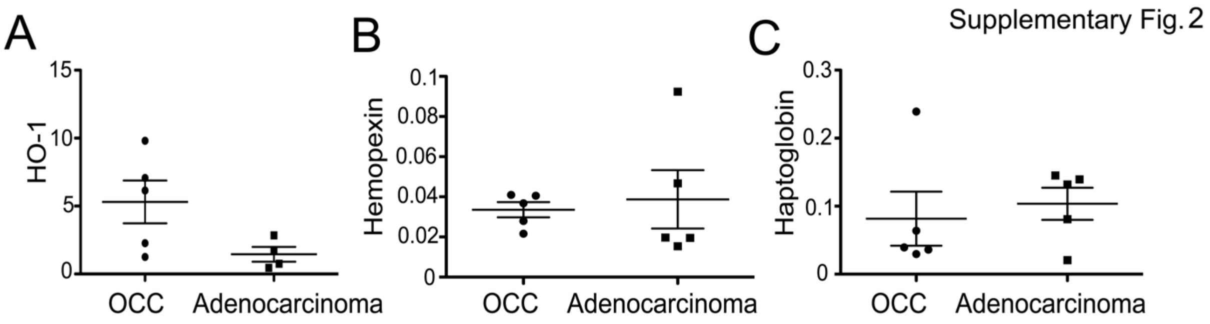

Figure S2: Geo Profile comparison of the relative expression of *Hmox1, Hx* and *Hp* in the ovarian clear cell-like cancer (OCC) or adenocarcinoma of ovary cell lines.

Supplementary File S1 (original blots).

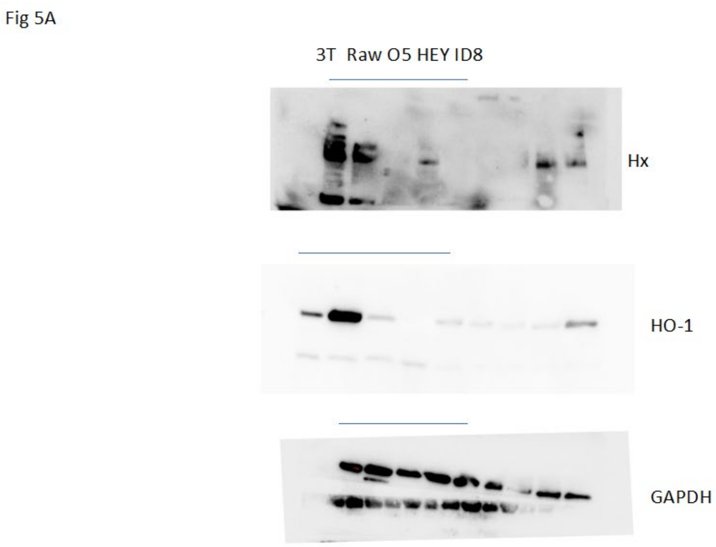

Fig. 5B

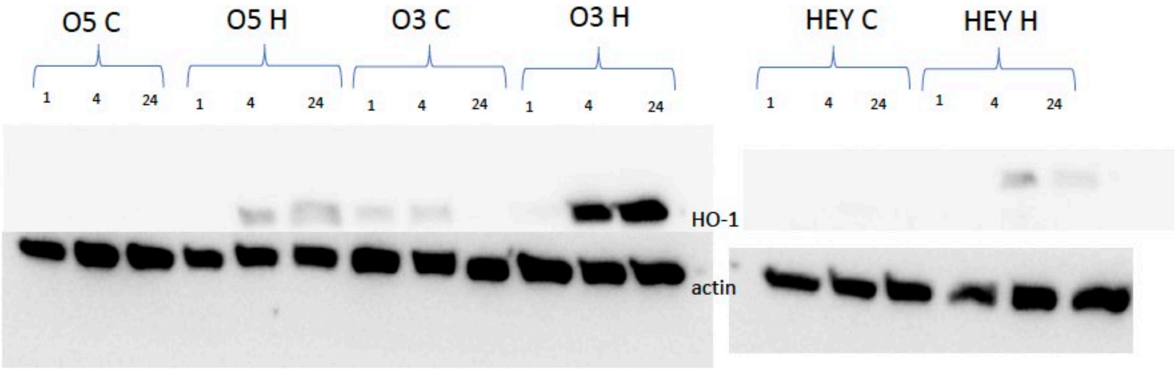

Fig 5C

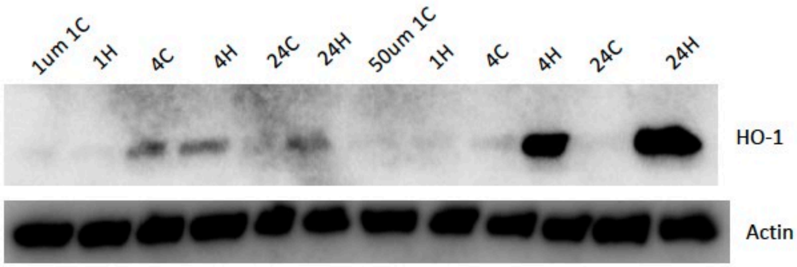

HEY cells
